# The Potential Role of Genomic Medicine in the Therapeutic Management of Rheumatoid Arthritis

**DOI:** 10.3390/jcm8060826

**Published:** 2019-06-10

**Authors:** Marialbert Acosta-Herrera, David González-Serna, Javier Martín

**Affiliations:** Institute of Parasitology and Biomedicine López-Neyra, CSIC, Av. del Conocimiento 17. Armilla, 18016 Granada, Spain; sna.david@ipb.csic.es (D.G.-S.); javiermartin@ipb.csic.es (J.M.)

**Keywords:** rheumatoid arthritis, pharmacogenomics, methotrexate, anti-TNF, personalized medicine

## Abstract

During the last decade, important advances have occurred regarding understanding of the pathogenesis and treatment of rheumatoid arthritis (RA). Nevertheless, response to treatment is not universal, and choosing among different therapies is currently based on a trial and error approach. The specific patient’s genetic background influences the response to therapy for many drugs: In this sense, genomic studies on RA have produced promising insights that could help us find an effective therapy for each patient. On the other hand, despite the great knowledge generated regarding the genetics of RA, most of the investigations performed to date have focused on identifying common variants associated with RA, which cannot explain the complete heritability of the disease. In this regard, rare variants could also contribute to this missing heritability as well as act as biomarkers that help in choosing the right therapy. In the present article, different aspects of genetics in the pathogenesis and treatment of RA are reviewed, from large-scale genomic studies to specific rare variant analyses. We also discuss the shared genetic architecture existing among autoimmune diseases and its implications for RA therapy, such as drug repositioning.

## 1. Introduction

Human diversity includes, among other things, differential responses to environmental stimuli and stress and differential drug metabolism and responses to treatments [1]. This diversity has been in part revealed since the completion of the Human Genome Project, which has improved our knowledge on the underlying biology of disease and its impact on healthcare. Since the completion of this project, a developing field called “personalized medicine” has aimed to adapt medical care to the genetic background of individuals, including diagnosis, therapeutic decision-making, health outcomes, and policy implications of clinical use (National Human Genome Research Institute (NHGRI), https://www.genome.gov/).

The most common variations in human DNA are single-nucleotide polymorphisms (SNPs), which are simple substitutions of one nucleotide for another at a given position [2]. These variants can map into gene coding or noncoding sequences of the genome, and the most studied to date have been biallelic SNPs [3]. These genome variations have been the objects of study of personalized medicine, which has revealed how subtle variations on the genome are responsible for large differences in health outcomes [1]. The main goal of these studies has been to detect associations between genetic variants and a trait or disease [4]. Under the common disease/common variant hypothesis, where common diseases are likely influenced by common genetic variants in the population, traditionally these studies have been focused on candidate gene association studies by comparing the allele frequency of the SNPs in affected and unaffected individuals. If the observed differences were not due to random chance, then they were considered to be associated with the disease or trait. Gene selection was based on the plausible mechanisms involved in disease pathogenesis [1]. At present and thanks to advances in genotyping technology and information on the haplotype structure of the genome, these studies are now directed toward the entire genome in so-called genome-wide association studies (GWAS). These studies are hypothesis-free (where no prior knowledge of the biological pathways is needed) and hypothesis-generating, as the new associations may pinpoint new molecular mechanisms never anticipated before. However, larger sample sizes are needed to fulfill the stringent threshold for statistical significance due to multiple testing adjustments (*p*-value < 5 × 10^−8^).

Since the completion of the first GWAS in 2005 [5], human genetic research has been a key player in the discovery of new biological pathways underlying complex diseases. Furthermore, genomic information may allow for the identification of patients with differential abilities to metabolize drugs, assess drug reactions, and eventually develop individualized treatments [6,7], which is the aim of the “treat-to-target” approach, where the therapeutic goal is to reach a state of disease remission or at least lower disease activity [8]. Many challenges continue to exist in the interpretation of GWAS findings. However, it has become fairly clear that drug development with genetic support from GWAS data is twice as likely to reach approval for its use as without this support (from phase I to approval in the different phases of drug development) [9]. In a recent paper by Nelson et al., the authors assessed publicly available GWAS results and combined them with the commercial Informa Pharmaprojects database. They found that those genes associated with a broad spectrum of human diseases were enriched in target genes for drugs approved in the United States or the European Union, highlighting the importance of the provided genetic knowledge in different drug mechanisms. The authors commented that this correlation may be explained by genes with prominent phenotypic changes that might be the most responsive to drug-induced alterations [9].

Interestingly, there have been several cases of genes associated with diseases that were effective drug targets. The most recognized examples have come from cholesterol metabolism: For instance, the gene *HMGCR*, which is associated with serum cholesterol levels [10], is a known target for statins. Additionally, loss of function mutations on the gene *PCSK9* have been described [11], and drugs targeting this gene have been developed to lower cholesterol levels [12,13].

## 2. Genetics and Therapy Development in Rheumatoid Arthritis

In the largest genetic study of rheumatoid arthritis (RA) conducted to date [14], the authors performed a three-stage transethnic meta-analysis in a total of 100,000 subjects of European and Asian ancestry by evaluating ~10,000,000 SNPs. Stage 1 revealed 57 associated loci, including 17 that had never been associated with the disease before. Afterward, the authors conducted a two-stage replication study for the suggestive loci (*p*-value < 5 × 10^−6^), and in a combined analysis of the three stages, they were able to identify 42 novel loci in the transethnic meta-analysis, increasing the total number of RA risk loci to 101. These loci represented a total of 377 associated genes, which were then prioritized. Interestingly, the authors assessed if the associated loci were useful for drug target validation and how approved drugs for other diseases could be linked to RA risk genes, evaluating not only the protein products of the RA-associated genes but also the protein that directly interacts with them in a protein–protein interaction network. From this assessment, the authors found a significant overlap among approved drugs for the treatment of RA that targeted genes that were considered RA risk genes in genetic studies. Additionally, the authors assessed if approved drugs for other diseases might be linked to RA risk genes for drug repurposing and found, for instance, the cases of *CDK4* and *CDK6*, which are targets for different types of cancer treatment [15] and have been shown to weaken disease activity in animal models of RA [15]. All of the above evidence has shown how human genetic data have the potential to be integrated with other biological information to enable personalized treatments.

## 3. Pharmacogenomic Studies in Rheumatoid Arthritis

Personalized medicine offers fully customized drugs for each person, attending to different conditions (such as genetics), and pharmacogenomics may identify the individual patient’s signature, which could help guide treatment selection mostly based on an assessment of genomic variants associated with drug response [16]. In the case of RA, even though routine compounds are suitable for most patients, some of them may not have the desired effect when taking these drugs [17], and patients may remain with high disease activity and irreversible joint damage as a possible consequence [18]. Thus, biomarkers are needed that help us differentiate between good and bad responses to a specific treatment. Although great progress has been made in this field, the objective of finding predictive genetic biomarkers that clearly define the grade of response to specific treatments is still far away. The use of genetic variants as biomarkers that could predict the response to a specific treatment has several advantages, as these variants are stable and would remain unaltered, unlike changes in gene expression and epigenetics, which are highly dependent on the environment.

### 3.1. Genomic Predictors of Methotrexate

Methotrexate (MTX) is a disease-modifying antirheumatic drug (DMARD) used as first-line therapy in RA [19], and great efforts have been directed toward finding predictive biomarkers of MTX response. These investigations have been centered on analyzing genes involved in the key molecular pathways affecting MTX absorption and metabolism, such as cytokine production, drug transport, or nucleotide synthesis. Most of these studies have been carried out in common variants, and the strongest and most replicated association discovered was for the solute carrier family 19 member 1 (*SLC19A1*) gene, a transport carrier that allows MTX to enter cells. In this sense, several studies have reported the association of rs1051266 with intracellular MTX levels. Indeed, a recent meta-analysis that included 12 studies confirmed the association of this polymorphism with MTX treatment response [20]. The methylene tetrahydrofolate reductase (*MTHFR*) gene has also been one of the most studied genes, as the encoded enzyme is key in the MTX pathway (C677T and A1298C are the most commonly studied SNPs associated with MTX response and toxicity [21,22]): However, there were conflicting results in two meta-analyses [23,24]. Another commonly studied polymorphism is the 347 C/G in the 5-aminoimidazole-4-carboxamide ribonucleotide formyltransferase (*ATIC*) gene, which was recently meta-analyzed in six studies that confirmed its association with responses to and the toxicity of MTX [23]. Although many other SNPs have been associated with MTX response and toxicity in RA patients, most of them could not be replicated, and the studies had a low sample size.

To date, two GWAS have been performed involving MTX activity: Senapati et al. [25] suggested multiple novel risk loci involved in thymidylate synthase (*TYMS*) regulation, and Taylor et al. [26] conducted the largest GWAS on MTX response, including 1424 early RA patients and finding a suggestive association in neuregulin 3 (*NRG3*). However, the authors were not able to replicate their findings. Thus, the existence of predictive models, including several SNPs from different genes associated with MTX response, could help in the creation of specific treatments associated with pharmacogenomics. In this regard, the clinical pharmacogenetics model of response to MTX (CP-MTX) combined clinical data and genotypes from four SNPs in MTX-relevant genes (*MTHF1D rs2236225*, *ATIC rs2372536*, *AMPD1 rs17602729*, and *ITPA rs1127354*). This model has been validated in independent samples, and its benefits and costs have been addressed in informing MTX prescription [27,28,29,30]. However, it is expected that many more genes and many more variants with modest effects contribute to response to treatment, and therefore the model could be further improved by including other clinical variables and updating the list of associated genetic variants. 

The major RA susceptibility region corresponds to the human leukocyte antigen (HLA) locus, concretely the *HLA-DRB1* shared epitope, which is associated with a more severe disease [31]. In this regard, there have been conflicting results regarding the association of the shared epitope with a lower MTX efficacy in monotherapy [32,33]. An additional *HLA-DR* locus, *HLA-DRB4*, is present in nearly all haplotypes containing the strongest associated *HLA-DRB1* alleles [34]. In this sense, a recent study compared MTX responders and nonresponders after stratification for *HLA-DRB4* expression, highlighting that response to MTX is characterized by preponderant innate and adaptive immune activation, respectively [35].

### 3.2. Genomic Predictors of Tumor Necrosis Factor (TNF) Inhibitors

Biomarkers able to predict responses to biological drugs have received lots of attention. In this line, tumor necrosis factor inhibitors (TNFis) remain the most commonly prescribed first-line biologics, even when these drugs are ineffective in up to 30% of patients [36]. Thus, more than 40 candidate gene studies and 6 GWAS regarding the response to TNFi have been performed to date [37,38]. One of the most commonly studied SNPs is G308A in the tumor necrosis factor (*TNFA*) gene, which has been associated with increased efficacy of adalimumab, infliximab, and etanercept [39,40]. In addition, Krintel et al. [41] suggested an association with *PDE3A–SLCO1C1*, and their results were replicated in a Spanish independent sample, reaching genome-wide significance [42]. Other SNPs linked to clinical responses in anti-TNF therapy are located on protein tyrosine phosphatase receptor type C (*PTPRC*): This has been consistently replicated in independent study samples [43,44,45]. Other studies have assessed the involvement of the fragment C gamma receptor (*FCGR*) [46], the TNF receptor superfamily 1B (*TNFRSF1B*) [47], and mitogen-activated protein kinase 14 (*MAPK14*) [40] in associations with TNFi response.

There have been many research efforts regarding the HLA region and its implication for TNFi therapies, as it happens with MTX response. One of the first associations observed involved two *HLA-DRB1* alleles encoding the shared epitope, including * 0101 and * 0404, in response to etanercept [48]. Subsequent studies confirmed the association of this locus with anti-TNF treatments, specifically with amino acid positions 11, 71, and 74 [31]. Furthermore, another study identified polymorphisms within the nonclassical *HLA-E* gene associated with clinical outcomes of anti-TNF therapy in female RA patients [49]. Unfortunately, the majority of studies that have been performed to date regarding pharmacogenetics of anti-TNF therapies have revealed inconsistent results, and very few of them have been robustly replicated [50,51]. This lack of replicability might be due to a lack of consensus on the criteria to differentiate the good versus bad responders [51].

Interestingly, a recent study by Sieberts et al. [52] showed that common SNP information did not improve significantly predictive models in contrast to other clinical information. They performed a community-based open assessment and tested a wide range of state-of-the-art modeling methodologies. However, the authors acknowledged some limitations when the number of risk loci was in the order of hundreds or when heritability was better explained by rare variations or copy number variants, which could be the case for TNFi response.

### 3.3. Other Genomic Predictors

DMARDs such as MTX and biologic agents are the drugs mainly used to treat RA. Nevertheless, there are other concomitant therapies used to reduce inflammation and relieve pain, including steroids and nonsteroidal anti-inflammatory drugs (NSAIDs). In this regard, two studies observed a better response to the combination therapy of MTX and glucocorticoids in RA patients carrying the mutant allele of the C3435T SNP of the multidrug-resistance 1 (*MDR1*) gene [53,54]. In addition, a subsequent study observed that carrying the SNP G2677A/T of the *MDR1* gene was significantly associated with response to glucocorticoid treatment [55].

On the other hand, like other DMARDs, one-third of patients fail to respond to MTX treatment, either because of inefficiency or adverse events. In those cases, leflunomide represents a potential drug to replace MTX as a treatment [19]. Pharmacogenetic studies have indicated an impact of the CYP1A2*1F mutation of the cytochrome P450 family 1 subfamily A member 2 (*CYP1A2*) gene in leflunomide toxicity [56]. Another study observed that rs3213422 of the dihydroorotate dehydrogenase (*DHODH*) gene was associated with leflunomide toxicity and therapeutic effects [57]. Finally, estrogen receptor gene SNPs could influence the response to leflunomide therapy in females [58].

## 4. Genetic Studies and Rare Variants

International collaborative efforts have enabled the recruitment of unprecedented sizes of study participants: However, despite the increase in statistical power, today many challenges still exist in the interpretation of findings from GWAS, which cannot account for much of the heritability of diseases (the “missing heritability” paradigm) [59]. The possible contribution of rare variants (minor allele frequency (MAF) < 0.5%) with larger effects on this missing heritability has been much discussed, and this may require composite association tests of overall “mutational load” in cases and controls. These genetic variations are not well captured by genotyping arrays. Therefore, whole-exome (WES) and whole-genome sequencing (WGS) is the technology of choice: It generates millions of sequence reads in parallel, increasing the speed and generated volume of data [59]. This technology will provide a deeper characterization of genetic variants within the entire frequency spectrum and their relationship to disease susceptibility. 

The impact of rare human variations on complex diseases is still limited, but for most traits, there is an inverse relationship between allele frequency and effect size, with the rarer alleles being those with a higher odds ratio, resembling those in Mendelian disorders [6]. A limited number of studies have assessed these rare genetic variations with success in RA. Li et al. [60] performed a WES study on 124 subjects from an Asian population. The authors identified genes enriched in deleterious variants using a gene burden test that were involved in innate immunity pathways and contributed to the risk of RA in a Han Chinese population. In the case of Bowes et al. [61], the authors utilized an interesting approach by exploiting low-frequency GWAS data by partitioning the data into gene-centric bins and collapsing their genotypes into a single count. The authors were able to prioritize signals mapping to *TNFAIP3*, a known RA risk gene, with replicable results in independent study samples. This study highlighted a previously known hypothesis where genes harboring common variants also harbored rarer variants with larger effects that had not been captured in previous GWAS [59]. To further confirm this hypothesis, Diogo et al. [62] assessed the contribution of rare and common variants in candidate genes from GWAS on RA by deep-sequencing their protein-coding regions. The authors exome-sequenced 25 RA risk genes from GWAS and found an aggregation of rare variants in *IL2RA* and *IL2RB*. They additionally assessed the aggregate contribution of low-frequency and common coding variants and observed an enrichment of coding variants with a nominal signal of association. The authors finally acknowledged the need for increased sample sizes to comprehensively identify variants distributed across the allele frequency spectrum associated with RA.

Interestingly, Eyre et al. [63] evaluated if previously associated linkage peaks were enriched with rare variants. They found that the distributions of rare variants differed significantly among regions showing linkage evidence, but that this effect depended on associations in the HLA region. Along the same line, Bang et al. [64] performed targeted exome sequencing in Korean RA patients. They comprehensively analyzed 10,588 variants of 398 genes and identified 13 nonsynonymous variants with nominal associations and 17 genes with nominal burden signals. However, the authors did not find a significant enrichment of coding variants associated with RA. As mentioned before, there has been little success in confidently identifying rare variants associated with RA, and in the majority of cases, studies have been performed on biological candidate genes. Only completely resequencing the whole exome or genome will eventually pinpoint novel genes harboring rare variants and significantly contribute to the proper assessment of RA heritability.

Interestingly, rare variants have also been evaluated in responses to treatment, as in the case of TNFi. Cui et al. [65] sequenced the coding region of 750 genes in 1,094 RA patients treated with anti-TNF. The authors applied single-variant association, gene-based associations, and gene set analyses in TNF pathway genes: However, they were not able to identify rare and low-frequency protein-coding variants that significantly contributed to anti-TNF treatment response. 

## 5. Shared Genetics in Autoimmunity and Drug Repurposing

Autoimmune disorders, such as RA, are very heterogeneous and share symptoms, risk genes, comorbidities, and familial aggregation, suggesting a common genetic architecture that is extensively recognized in autoimmunity [66,67]. Several studies have revealed these shared genetics through simple comparisons of the associated genes [68], as was the case in a study conducted by our group, where González-Serna et al. [69] considered the association of a rare variant in the *TNFSF13B* gene with RA and replicated this association in systemic lupus erythematosus (SLE) patients. *TNFSF13B* encodes the (B-cell activating factor) BAFF cytokine, which is essential for B-cell homeostasis and the regulation of B-cell maturation, differentiation, and survival [70]. The assessed risk variant is functional and results in a shorter transcript that escapes microRNA inhibition, leading to an increase in the production of the BAFF cytokine. Additionally, it has been observed that this variant is strongly associated with high levels of total IgG and IgM and with reduced monocyte counts [71]. Our reported association with RA highlights the BAFF variant as a common genetic risk factor in autoimmunity. Interestingly, belimumab is a monoclonal antibody targeting human BAFF and was the first targeted therapy approved for SLE [72,73,74], highlighting the potential for drug repurposing for genetically related conditions.

Recently, a combination of large-scale studies including different phenotypes has proven to be a very useful tool in the identification of shared genetic risk variants and shared pathways involved in these diseases in a systematic fashion [75,76,77]. The first example is a meta-GWAS that combined 10 pediatric autoimmune diseases and revealed new shared loci with immunoregulatory functions [78]. Another big study by Ellinghaus et al. [79] combined immunochip data from five chronic inflammatory diseases and also found shared genetic loci in seronegative conditions, such as ankylosing spondylitis, psoriasis, primary sclerosing cholangitis, Crohn’s disease, and ulcerative colitis. Furthermore, two recent studies by Marquez et al. [80] and Acosta-Herrera et al. [81] assessed the genetic overlap in autoimmune diseases by combining Immunochip and GWAS data, respectively. The authors identified shared risk variants in autoimmunity and common biological mechanisms and suggested novel genes as drug targets as well as promising candidates for drug repurposing. These studies highlighted how a combination of different related phenotypes can contribute to the determination of causal variants in disease and might help in the establishment of personalized treatments.

## 6. Future Perspectives

Most RA treatments are nonspecific and are based on a trial-and-error approach. Ineffective treatments affect the quality of life of patients, increasing the probability of adverse events and the eventual development of greater disabilities. It is therefore crucial to develop mechanisms to deliver the right drug to the right patients, which is the ultimate goal of precision medicine. In this sense, a small number of associated loci have been consistently replicated regarding treatment response: One of the reasons might be the lack of statistical power of these assessments. RA is a complex and heterogeneous disease, and therefore the response to treatment in RA patients might be influenced by multiple effects of many genetic variants with moderate effect sizes. Thus, larger studies with proper statistical power and more homogeneous classifications of phenotypes are needed to validate previous findings.

Moreover, response to treatment is normally measured with several composite scores, including the disease activity score (DAS-28), American College of Rheumatology, or EULAR response criteria, hindering the possibility of validating the results. Well-described and homogenous measures of responses are critical in pharmacogenetic studies. Additionally, most studies have investigated common genetic variations in treatment response, and a broader assessment of the frequency spectrum might be necessary.

Establishing the association of genes should continue with discoveries of the functional implications of such associations. As commented before, genetic variants alone do not fully explain the risk of suffering the disease, making complementary strategies necessary, including transcriptomic and epigenomics approaches: For example, Plant et al. [82] identified differentially methylated positions on DNA as biomarkers of response to TNFi therapy. Additionally, Spiliopoulou et al. [83] showed that the CD39 and CD40 pathways could be relevant to targeted drug therapy by evaluating the relations of TNFi responses with locus-specific scores, constructed from GWAS data. 

Finally, the integration of all of this information with clinical and environmental data will eventually help us to identify the biological mechanisms underlying the development of the disease, to establish accurate biomarkers for patient stratification, and to tailor treatment to genetic architecture, increasing the probability of obtaining an adequate response to a particular drug and eventually achieving disease remission.
